# Burden of Financial Hardship Among Breast Cancer Survivors in Maharashtra, India

**DOI:** 10.7759/cureus.61625

**Published:** 2024-06-03

**Authors:** Abhilash Patra, Rebecca deSouza, Shona Nag, Hira B Pant, Varun Agiwal, Nirupama A Y, Yashaswini Kumar, GVS Murthy

**Affiliations:** 1 Epidemiology and Public Health, Indian Institute of Public Health, Hyderabad, IND; 2 Oncology, Sahyadri Group of Hospitals, Pune, IND; 3 Data Management and Biostatistics, Indian Institute of Public Health, Hyderabad, IND

**Keywords:** india, survivorship, breast cancer, financial hardship, burden

## Abstract

Introduction: Improved breast cancer treatments have increased survival rates, but prolonged and costly therapies strain survivors financially. This study addresses the dearth of research on financial difficulties among breast cancer survivors (BCS) in India.

Methods: A mixed-methods study was employed; we assessed financial hardship (FH) using the Comprehensive Score for Financial Toxicity-Functional Assessment of Chronic Illness Therapy (COST-FACIT), a validated 12-item questionnaire. The minimum score represents FH (FH was categorized based on scores <27).

Results: Out of 80 surveyed BCS, 60% experienced FH and had a median age of 48 years (40.5-56.5 years). Factors such as occupation, education, income, expenditures, insurance coverage, and impact on savings exhibited significant associations with FH. With only one-third having health insurance and 43.8% self-funding treatment, this research sheds light on the urgent need for targeted support and policies to alleviate the financial burdens faced by BCS in the Indian context.

Conclusion: Financial hardship harms the mental and physical health of BCS. Collaborative efforts among policymakers, healthcare professionals, and insurers are crucial to establishing a compassionate healthcare system that addresses both immediate health and long-term financial concerns.

## Introduction

Cancer incidence is rapidly increasing in India. Studies report that there will be a 12% increase in the incidence of cancer by 2025 [1]. The burden of breast, cervical, and colorectal cancers is greater in females, whereas the burden of oral, stomach, and esophagus cancers is greater in males [1]. Cancer treatment has remarkably improved, thereby improving patient outcomes; however, those improvements have come at an increasing cost [2, 3]. Moreover, with improvements in treatment and early detection, breast cancer prevalence and survivors's burden are gradually increasing [4]. With a gradual increase in breast cancer survivors (BCS), life spent with cancer-associated complications and disability has also increased [5].

Increased disability and complications led to productivity and earning loss, which subsequently led to catastrophic healthcare expenditure [5]. Studies have reported the out-of-pocket expenditure (OOPE) of cancer has increased from Rs 2,895/- and Rs 52,393/- for outpatient and inpatient care, respectively, as compared to other chronic diseases [6]. Multiple studies reported OOPE in the inpatient care of cancer patients, whereas none focused on survivors [7, 8].

Lack or insufficiency of money or resources essential to fulfilling basic needs of life such as food, clothing, shelter, and medical care is commonly understood as financial hardship (FH) [9]. Studies conducted in developed countries reported FH among cancer survivors to be 28% to 48% [10]. The cause of FH was reported to be costs associated with cancer care, loss of employment, low income, and medical debt [11]. Also, increased financial burden has been reported to affect cancer treatment choice, compliance, and outcome [12]. Those survivors who report FH have been shown to delay, discontinue, or forgo care, leading to decreased treatment adherence and subsequently leading to poor quality of life [13-15].

Multiple studies have reported FH and distress among BCS in developed countries [13,16-21]. However, there is very limited research reported in low- and middle-income countries (LMICs) [22]. A systematic review conducted by Boby et al. reported financial toxicity among patients with cancer in India and suggested the need for urgent mitigation strategies [23]. However, information concerning BCS is scarce [24]. Also, as far as our knowledge goes, there is no information on FH among BCS in India. Hence, we examined FH among BCS in Maharashtra, India. The main objective was to examine the burden of FH and the causes and repercussions among BCS.

## Materials and methods

A cross-sectional mixed-method study consisting of two phases, the first phase includes a quantitative questionnaire involving demographic details along with a validated Comprehensive Score for Financial Toxicity-Functional Assessment of Chronic Illness Therapy (COST-FACIT) [25]. The second phase includes a focus group discussion (FGD) to understand the causes and effects of financial hardship among BCS who attended the 11th Indian Breast Cancer Survivorship Conference held on October 1st and 2nd, 2023, organized by the Nag Foundation in collaboration with other local bodies in Goa. The conference was attended by 150 BCS from different parts of Maharashtra and a few from Goa. For the quantitative method, all the BCS were informed about the study during the registration process of the conference, and those who consented to participate were included. For FGD, seven to 10 breast cancer survivors were approached and those who consented were included in the qualitative method. 

Data collection tools and process

All the BCS who consented to participate were provided with a self-reported questionnaire, including demographic questions. The questionnaire also included a validated COST-FACIT [25]. The COST-FACIT is a validated 12-item questionnaire, with each item having a response scale similar to the five-point Likert scale. The score ranged from 0-44. The higher the score, the better the financial well-being. Each participant received a comprehensive explanation of every question in the survey form. It took approximately 15 minutes for each participant to complete the survey. To ensure data completeness, one researcher cross-checked the forms, and if any were found to be incomplete, participants were reached out to in person on the second day of the conference to provide the necessary information. Further, telephonic calls were used to complete the data.

An interview guide was developed that included open-ended questions related to FH, its causes, and its effects. Two interviewers were involved in conducting the FGD. Participants were informed about the time and venue for the discussion, and verbal consent was obtained from the participants. The discussion was continued until saturation (a similar and repetitive response from the participants) in response was achieved. The discussion was audio-recorded with the participant’s permission. Along with the recording, one of the interviewers took notes during the discussion.

Data analysis

A paper-based data collection process was involved, and further, the collected data were entered in Microsoft Excel (Microsoft Corp., Redmond, WA). The data were coded, and analysis was performed in Stata version 14 (StataCorp LLC, College Station, TX). Demographic data was reported in frequency, percentage, mean and standard deviation (SD), or median and interquartile range (IQR). Seven out of 12 items in the COST-FACIT were reversed, and individual items were summed up to obtain the total score. Further, the sum of the item scores was multiplied by the number of items on the scale and then divided by the number of items answered. This whole process produced the scale score. The higher the score, the better the financial well-being. Since there is no defined threshold for financial hardship screening using the COST-FACIT score, the median financial hardship score was further classified into two groups: those with a score of <27 and those with a score of ≥27 [26]. Survivors with <27 scores were considered to have financial hardship. A Shapiro-Wilk test along with a histogram was used to assess the normality of the data. The chi-square and Mann-Whitney U tests were applied to assess the association between financial hardship and demographic variables. Statistical significance of p <0.05 with a 95% confidence interval (CI) was considered.

A thematic framework was used to explore the causes and effects of FH faced by BCS. Themes were developed based on the frequent appearance of certain verbatims or phrases and whether the themes captured important aspects of the research questions. The recorded FGD was transcribed by listening several times to become familiar with the data. Transcripts were generated, translated, and double-checked by another researcher. Subsequently, codes were generated and color-coded using Microsoft Word (Microsoft Corp.), and all relevant codes were summarized into themes along with verbatim transcripts.

## Results

A total of 150 BCS attended the conference, of whom 80 belonged to Maharashtra and consented to participate in the study. The median age of the survivors was 48 years, with an IQR of 40.5 to 56.5 years. The majority of the survivors were Hindu (78.75%) and from the general category (71.25%). More than four-fifths of the survivors were married (83.75%), and a majority of them were housewives (51.25%). More than three-fourths of the survivors had studied in college or above (77.50%) and were diagnosed with breast cancer within five years (78.75%). Nearly half of the survivors were undergoing some kind of breast cancer treatment (48.75%), and more than 75% of them were non-metastatic. The average household monthly income of the survivors was Rs 40,000/-, with IQR ranging from Rs 14000/- to Rs 100,000/-. More than three-fifths of the survivors did not have insurance, and among them, 43.75% bore their treatment expenses by themselves, whereas 17.50% of them received support from non-governmental organizations (NGOs), and the rest 5% of them took out loans to cover their breast cancer treatment costs (Table 1).

**Table 1:** Demographic characteristics of breast cancer survivors IQR: interquartile range; NGO: non-governmental organization

Demographic characteristics	n (%)
Age (in years), median (IQR)	48 (40.5, 56.5)
Religion	
Hindu	63 (78.75%)
Muslim	4 (5.00%)
Christian	13 (16.25%)
Caste	
General	57 (71.25%)
Scheduled Caste	9 (11.25%)
Scheduled Tribe	1 (1.25%)
Other Backward Class (OBC)	13 (16.25%)
Marital status	
Unmarried	4 (5.00%)
Married	67 (83.75%)
Divorced/separated/widowed	9 (11.25%)
Household monthly Income (in Rupees), median (IQR)	40,000 (14,000, 100,000)
Occupation	
Government employee	11 (13.75%)
Private employee	20 (25.00%)
Housewife	41 (51.25%)
Business	8 (10.00%)
Education	
Primary	3 (3.75%)
Secondary	15 (18.75%)
College and above	62 (77.50%)
Time since diagnosis (in years)	
>5 years	17 (21.25%)
≤5 years	63 (78.75%)
Under treatment	
Yes	39 (48.75%)
No	41 (51.25%)
Disease status	
Metastatic	19 (23.75%)
Non-metastatic	61 (76.25%)
Have insurance	
Yes	27 (33.75%)
No	53 (66.25%)
Expenditure incurred	
Government insurance	9 (11.25%)
Private insurance	18 (22.50%)
Support from NGOs	14 (17.50%)
Took loan	4 (5.00%)
Self	35 (43.75%)
Savings affected	
Yes	67 (83.75%)
No	13 (16.25%)

More than 80% of the survivors reported that their savings were impacted due to breast cancer treatment, and 60% (95% CI, 49%-70%) of the BCS faced FH. Breast cancer survivors who had FH had an average household monthly income of Rs 21,000/- as compared to those who did not have FH, who had an average household monthly income of Rs 100,000/- and were significantly associated (p <0.001). The majority of the survivors who had FH were housewives (62.5%) and private employees (27.08%) (p <0.05). Financial hardship was also significantly associated with education, incurred expenditures, insurance, and the impact of savings (Table 2).

**Table 2:** Association of financial hardship with demographic characteristics of breast cancer survivors ^a^Mann-Whitney U test; ^b^Chi-square Test; ^#^p-value <0.05 considered statistically significant; IQR: interquartile range; NGO: non-governmental organization

Demographic characteristics	With financial hardship (n=48) (n,%)	Without financial hardship (n=32) (n,%)	p-value
Age (in years), median (IQR)	51 (42,60)	46 (40, 44)	0.29^a^
Religion			
Hindu	38 (79.17)	25 (78.13)	0.75^b^
Muslim	3 (6.25)	1 (3.13)	
Christian	7 (14.58)	6 (18.75)	
Caste			
General	33 (68.75)	24 (75)	0.42^b^
Scheduled Caste	7 (14.58)	2 (6.25)	
Scheduled Tribe	0 (0)	1 (3.13)	
Other Backward Class (OBC)	8 (16.67)	5 (15.63)	
Marital status			
Unmarried	3 (6.25)	1 (3.13)	0.39^b^
Married	38 (79.17)	29 (90.63)	
Divorced/Separated/Widowed	7 (14.58)	2 (6.25)	
Household monthly income (in Rupees), median (IQR)	21000 (10000, 45000)	100000 (50000, 200000)	0.000^a#^
Occupation			
Government employee	3 (6.25)	8 (25)	0.02^b#^
Private employee	13 (27.08)	7 (21.88)	
Housewife	30 (62.5)	11 (34.38)	
Business	2 (4.17)	6 (18.75)	
Education			
Primary	3 (6.25)	0 (0)	0.000^b#^
Secondary	15 (31.25)	0 (0)	
College and above	30 (62.5)	32 (100)	
Time from diagnosis in years			
>5 years	9 (18.75)	8 (25)	0.5^b^
≤5 years	39 (81.25)	24 (75)	
Under treatment			
Yes	26 (54.17)	13 (40.63)	0.23^b^
No	22 (45.83)	19 (59.38)	
Disease status			
Metastatic	10 (20.83)	9 (28.13)	0.45^b^
Non-metastatic	38 (79.17)	23 (71.88)	
Have insurance			
Yes	10 (20.83)	17 (53.13)	0.004^b#^
No	38 (79.17)	15 (46.88)	
Expenditure incurred			
Government insurance	4 (8.33)	5 (15.63)	0.006^c#^
Private insurance	6 (12.50)	12 (37.50)	
Support from NGOs	13 (27.08)	1 (3.13)	
Took loan	3 (6.25)	1 (3.13)	
Self	22 (45.83)	13 (40.63)	
Savings affected			
Yes	47 (97.92)	20 (62.5)	0.000^b#^
No	1 (2.08)	12 (37.5)	

Thematic analysis

A total of eight BCS consented to participate in the FGD. Below are the challenges faced by the BCS.

Financial Challenges

The majority of participants had lost their jobs due to a breast cancer diagnosis. Few had limited insurance coverage, leading to a personal financial burden. All of them had to take out loans at a certain point in time during their treatment to cover the cost.

Unemployment

"I was working earlier, but when I had cancer, I had to leave my job, so the source of income got lost, and we had only one person as the bread-earner, which led to managing my cancer treatment bills and my home," said one participant.

One participant said, "When I was diagnosed, a month after that my husband lost his job as he was frequently coming with me to the hospital, so at that time the burden of finance and daily expenses came all together."

Borrowed Money

A participant said, “After my breast cancer treatment was over, as I had no insurance, I took a loan, so I had to pay them. But after a few years, I got to know that I had cancer in my uterus; at that time, I had to sell my gold to get my treatment done.”

Treatment (Before and After)-Related Financial Issues

The cost of specific treatments, such as comorbidities that occurred post-chemotherapy and radiation therapy, was not covered by insurance. The majority of them also stated that you can avail of insurance only when you are hospitalized; however, costs involving preparation before treatment, out-patient costs, and complications post-treatment were not covered, leading to financial strain in their day-to-day lives. Challenges in managing frequent follow-ups and unforeseen health issues arising after cancer treatment also led to financial strain.

Financial Insufficiency

“The only salary that used to come was from my husbands, and when my children were studying, it was a dent in our savings, and at that time they asked me to go for radiation therapy, which I skipped for a while," said one participant.

Discontinued Treatment

“I am suffering from osteoarthritis. I had knee surgery, which was not successful. I have skipped my follow-up consultations because of financial distress. As the consultation fees and other costs are not covered by insurance, I had to gather the amount to go for follow-up. If doctors call me after two months, I go after five months”, said one participant.

Insurance Disparities

All the participants stated that out-patient costs, implant costs, the cost of plastic surgery, rehabilitation costs, and the cost of side effects that cancer patients face post-chemotherapy were not included in the insurance. Certain medicines, such as Herceptin, were also not included in the insurance scheme, which the patients need for the long term.

Insurance Coverage and Disparities

A participant said, “If you have side effects, those are not covered under insurance. Certain chemotherapy medications, such as Herceptin, are not covered by insurance. However, these small costs for a longer duration led to financial distress in breast cancer treatment. Also, the rehabilitation costs, such as treatment costs for lymphedema or if you want to buy a prosthesis, all those things are not covered by insurance.”

Another said, “Do not discriminate against us based on the disease; treat cancer as any other chronic illness where insurance is covered. We receive calls from insurance companies, but then they shut the phone once they get to know we are cancer survivors.”

“I have a heart problem, and I don’t have insurance; they want me to have surgery, which will cost more than Rs 9 lakh. I don’t have that kind of money, so I said to the doctor, when my tickle (heart) stops, I will be done,"said one participant.

Information and Awareness

Participants reported that insufficient and unclear information was provided about the financial implications of cancer treatment, and they also said there was limited awareness among healthcare professionals about patients' financial distress. There was also a need for financial knowledge for cancer patients. All the participants perceived a need for a financial counselor in hospitals to guide patients and insurance companies and to consider cancer as any other chronic illness.

Financial Literacy

“They don’t have any clue. They are not interested in our financial distress," said one participant.

Another participant said, "There is no information on the entire treatment; it’s like you go for surgery, then they give you the estimate, then your next step is chemotherapy, then they give an estimate for chemotherapy, then similar for radiation. Then, in between, you need injections post-chemotherapy, and then you need port insertion. I had to go for two surgeries for that, as no one informed me regarding port insertion. Then I had a chemo leak, and for that, I went for another treatment. Then you do a blood test. And then I had 10 years of hormone replacement therapy (HRT); no one financed me for that.”

"There should be a department along with a cancer department. As doctors focus on the treatment aspect and manage the psychological aspect of the patient, this department can focus on giving financial knowledge to the patient regarding the treatment cost. There should be someone who can be an advisor to provide information regarding the treatment cost of the cancer," said a participant.

## Discussion

The continued rise in the cost of cancer care in India over the past few years and its impact have driven the need to assess the FH associated with BCS [23]. In this study, we identified and summarized the prevalence of FH among BCS in Maharashtra, India. We found that more than half of the BCS had FH, which corroborates the findings of the study conducted by Ell et al. [27]. A mixed-methods study conducted by Alexander et al. among BCS in India reported financial insufficiency and the sale of assets as some of the major causes of FH, which substantiates our qualitative findings [28]. In our study, the majority of the survivors paid from their pockets, and the majority of the survivors who had private and government insurance also faced financial hardship. This brings to light the disparity faced by cancer survivors post-completion of their treatment to receive insurance. Our study also suggests that BCS do not have adequate information on survivorship care, treatment plans, and costs. Also, the decision to undergo certain treatments depends on their socio-economic condition.

Increased out-of-pocket expenditure and the high prevalence of FH among BCS bring to the attention an urgent need for greater investment in cancer survivorship care, along with increasing public investment in cancer care and policies. Also, the need to increase financial literacy and awareness of insurance is the need of the hour. Focus also needs to be given to the comorbidities that cancer survivors undergo and the financial implications that revolve around the screening, treatment, and rehabilitation of those comorbidities.

Inadequate insurance coverage emerged as a challenge for BCS, as qualitative interviews reported that they do not have enough insurance to cover certain treatment costs or are not covered by insurance. Policymakers can make an impact by expanding insurance coverage beyond cancer treatment, including outpatient treatment costs and costs associated with complications that do not require hospitalization. Also, insurance plans can be designed to promote equity in costs among individual insurance plans, such as value-based designs, which can help reduce cost disparities across BCS.

The limitations of this study include a small sample size and reliance on the experiences of women in the state of Maharashtra, India, which may restrict the generalizability of the findings. The analysis lacks a comprehensive list of demographic and clinical characteristics that could contribute to FH. However, these findings serve as a preliminary exploration of potential characteristics for future studies to investigate. The analysis falls short of identifying contributing factors, highlighting the need for future research to delve into these aspects and offer deeper insights into FH, ultimately leading to potential solutions.

## Conclusions

Financial hardship harms the mental and physical health of BCS. Collaborative efforts among policymakers, healthcare professionals, and insurers are crucial to establishing a compassionate healthcare system that addresses both immediate health and long-term financial concerns. Future studies can focus on developing interventions to improve financial literacy among cancer patients and empower them. The introduction of financial navigators not only enhanced access and support for patients but also decreased both patient and health system costs. Therefore, prioritizing the implementation of financial navigators in both private and government hospitals holds the potential to reduce the financial hardship experienced by cancer patients.
